# Targeting Src Family Kinases Inhibits Bevacizumab-Induced Glioma Cell Invasion

**DOI:** 10.1371/journal.pone.0056505

**Published:** 2013-02-14

**Authors:** Deborah Huveldt, Laura J. Lewis-Tuffin, Brett L. Carlson, Mark A. Schroeder, Fausto Rodriguez, Caterina Giannini, Evanthia Galanis, Jann N. Sarkaria, Panos Z. Anastasiadis

**Affiliations:** 1 Department of Cancer Biology, Mayo Clinic, Jacksonville, Florida, United States of America; 2 Department of Pathology, Mayo Clinic, Rochester, Minnesota, United States of America; 3 Department of Oncology, Mayo Clinic, Rochester, Minnesota, United States of America; 4 Department of Radiation Oncology, Mayo Clinic, Rochester, Minnesota, United States of America; Seoul National University, Republic of Korea

## Abstract

Anti-VEGF antibody therapy with bevacizumab provides significant clinical benefit in patients with recurrent glioblastoma multiforme (GBM). Unfortunately, progression on bevacizumab therapy is often associated with a diffuse disease recurrence pattern, which limits subsequent therapeutic options. Therefore, there is an urgent need to understand bevacizumab's influence on glioma biology and block it's actions towards cell invasion.

To explore the mechanism(s) of GBM cell invasion we have examined a panel of serially transplanted human GBM lines grown either in short-term culture, as xenografts in mouse flank, or injected orthotopically in mouse brain. Using an orthotopic xenograft model that exhibits increased invasiveness upon bevacizumab treatment, we also tested the effect of dasatinib, a broad spectrum SFK inhibitor, on bevacizumab-induced invasion.

We show that 1) activation of Src family kinases (SFKs) is common in GBM, 2) the relative invasiveness of 17 serially transplanted GBM xenografts correlates strongly with p120 catenin phosphorylation at Y228, a Src kinase site, and 3) SFK activation assessed immunohistochemically in orthotopic xenografts, as well as the phosphorylation of downstream substrates occurs specifically at the invasive tumor edge. Further, we show that SFK signaling is markedly elevated at the invasive tumor front upon bevacizumab administration, and that dasatinib treatment effectively blocked the increased invasion induced by bevacizumab.

Our data are consistent with the hypothesis that the increased invasiveness associated with anti-VEGF therapy is due to increased SFK signaling, and support testing the combination of dasatinib with bevacizumab in the clinic.

## Introduction

Malignant glioma tumors (glioblastoma multiforme or GBM) are the leading cause of CNS tumor-related mortality. Two major factors underlie the poor clinical outcome of these tumors: the intense angiogenic activity of GBM and their aggressive invasion into surrounding normal brain tissue. Recently, anti-angiogenic therapy has emerged as an important avenue for the treatment of GBM [1]–[5]. Studies with the humanized monoclonal antibody bevacizumab (Avastin), which targets the pro-angiogenic factor VEGF, have demonstrated significant therapeutic benefit in patients with recurrent GBM [6]–[9]. In addition, a randomized phase II trial of bevacizumab versus the bevacizumab/irinotecan combination confirmed the activity of single agent bevacizumab in the recurrent GBM setting [10]. These data have generated significant excitement in the neuro-oncology community, and therapy with bevacizumab is becoming the treatment of choice for recurrent GBM patients. Unfortunately, tumor recurrence on anti-angiogenic therapy often is associated with increased tumor invasiveness, and a significant proportion of patients progress on bevacizumab with a diffuse or multi-focal tumor recurrence pattern that is associated with rapid clinical deterioration [11]. Thus, while bevacizumab can result in significant temporary patient benefit, there is an urgent need to understand how anti-angiogenic therapies influence basic tumor biology, as well as to develop novel strategies to overcome the pro-invasive effects of bevacizumab therapy.

Orthotopic xenograft models have been used previously to show the benefits of anti-angiogenic therapy. For example, the inhibition of VEGF/VEGFR interactions using neutralizing antibodies, anti-sense and retroviral strategies represses angiogenesis and the growth of human GBM cells in flank and orthotopic animal models [1]–[5]. However, these models also provide clues to the pattern of tumor recurrence seen in human patients. In a rat orthotopic model of human GBM, anti-VEGF therapy resulted in increased animal survival, decreased tumor vascularity, increased apoptosis, and decreased tumor growth, but also resulted in increased GBM cell infiltration and cooption of existing vasculature [1]. Similarly, Kunkel et al. reported that inhibition of neo-angiogenesis by systemic treatment with an anti-VEGFR2 specific monoclonal antibody decreased microvessel density and tumor cell proliferation, increased apoptosis and inhibited overall tumor growth [3]. However, they also reported a striking increase in tumor cell invasion, cooption of cerebral vasculature, an increase in distinct satellite tumor foci, and eventual leptomeningial spread [3]. Other recent studies also indicate that potent anti-angiogenic inhibitors reduce primary tumor growth, but promote tumor invasion and metastasis [12], [13]. The preclinical data suggest that increased tumor invasiveness is a major impediment to the efficacy of anti-angiogenic GBM therapy. These findings help to explain the resistance to these drugs seen in the clinical setting, and raise the question of how to best treat cancer patients with anti-angiogenic therapies in the future.

Mechanisms that underlie the other major factor in the poor clinical outcome of GBM, aggressive glioma cell invasion, are inadequately understood. One possible mechanism that promotes invasion is the activation of Src family kinases (SFKs). Several common molecular alterations in gliomas result in increased SFK activity, including amplification of the epidermal growth factor or the platelet-derived growth factor receptors, or upregulation of integrin receptors such as α_v_β_3_ and α_v_β_5_ [14], [15]. Proteomic profiling of phosphorylated/activated tyrosine kinases shows that Src is frequently activated in human GBM lines and primary tumors [16]. One consequence of SFK activation is increased GBM tumor cell motility and invasion [14], [15], [17]–[19]. For example, Lyn kinase is highly expressed in GBM tumors and its activation by PDGFR and α_v_β_3_ induces cell migration [14]. Similarly, the SFK Yes forms a complex with CD95 and the p85 regulatory subunit of PI3K to induce GBM cell invasion [20]. Conversely, the Src-mediated migration of T98G cells *in vitro* and *in vivo* is selectively inhibited by dasatinib [16], a potent multi-targeted kinase inhibitor that inhibits all SFKs [18], [21]. Interestingly, in Src deficient transgenic mice, tumor angiogenesis is decreased together with glioma invasion/infiltration [22]. Consistent with this, angiogenesis is increased following the expression of GFAP-v-Src in transgenic mice, which induces the formation of astrocytomas and hypervascularized glioblastomas [23]. Importantly, Src can be activated by tumor hypoxia and this activation promotes angiogenesis in part by the induction of VEGF expression [18], [24].

Activated SFKs affect cellular function via the tyrosine phosphorylation of key cellular substrates that are involved in cytoskeletal organization and the regulation of cell-cell adhesion or adhesion to the extracellular matrix [25], [26]. Src activation promotes cell migration by both disrupting cell-cell adhesion and by promoting the formation of integrin-mediated cell-extracellular matrix focal contacts [26], [27]. Src, as well as other SFKs, associates with and directly phosphorylates multiple targets within these adhesion complexes, including p120 catenin on Y228 [28], focal adhesion kinase (FAK) on Y861 [29], p130cas on Y410 [29], paxillin on Y118 [29], and Vav2 on Y172 [30]. Importantly, the migration and invasiveness of tumor cells can be markedly reduced by pharmacologic or molecular inhibition of SFK activity, FAK activity or p120 catenin [31]–[33]. The pro-migratory function of p120 catenin requires Rac1, a member of the Rho family of small GTPases which regulates actin polymerization and protrusion at the leading edges of migrating cells [34]. Activation of Rac1 occurs downstream of p120 interaction with the Rac1 exchange factor Vav2 [33], [35], [36]. Rac1 is also critical for p130cas-induced cell migration downstream of the Rac1 exchange factor DOCK180 [37], [38]. Thus, while not a direct target of SFK phosphorylation, Rac1 is nevertheless an important downstream SFK effector. Rac1 is also thought to be important in glioma cell migration and invasion [39], [40].

To explore the mechanistic importance of SFK activation for the invasiveness of GBM tumors we have examined a panel of serially transplanted human GBM lines grown either in short-term culture, as xenografts in mouse flank, or injected orthotopically in mouse brain [41]. Using this model, we show that the SFK-mediated tyrosine phosphorylation of key Src-substrates is elevated at the infiltrating edge of aggressive GBM tumors, and that this SFK activity is further induced by treatment with the anti-angiogenic agent bevacizumab. Importantly, treatment with the SFK inhibitor dasatinib significantly reduces GBM cell migration *in vitro*, and effectively blocks the bevacizumab-induced invasion and infiltration of orthotopically xenografted GBM10 cells.

## Results

**Figure 1:**
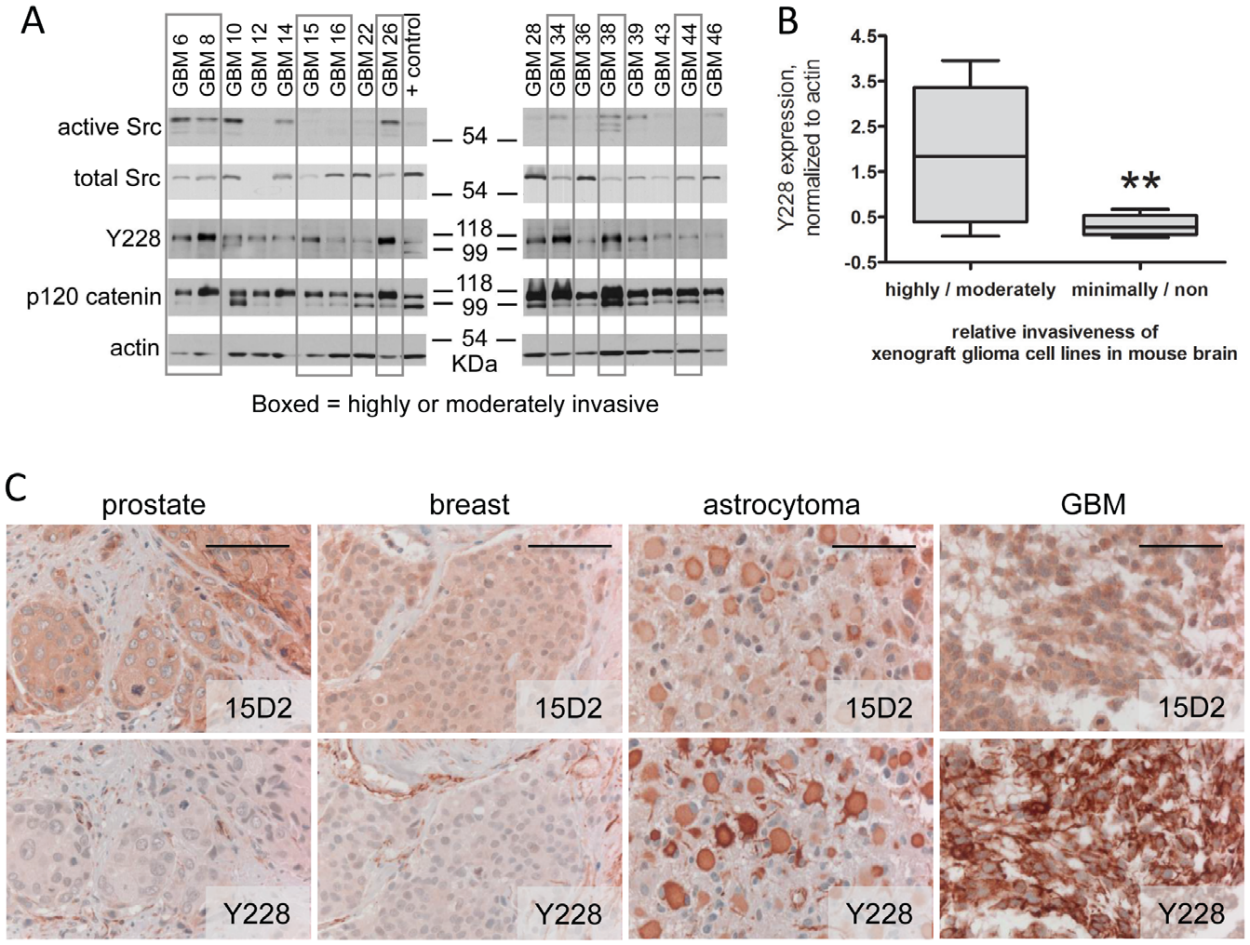
Activation of Src and p120 phosphorylation correlates with increased glioma invasiveness. **A.** Seventeen glioma cell lines propagated as xenografts in mouse flank were examined by western blot for expression of active (Y416-phosphorylated) and total Src and Y228-phosphorylated and total p120 catenin proteins. Actin is a loading control. The positive control lysate is from MDA231 cells. Xenograft lines were previously classified based on their relative invasiveness as highly or moderately invasive (data boxed in gray) or minimally or non-invasive (not boxed). **B.** The level of Y228-phosphorylated p120 catenin expression (relative to actin expression) as determined by Western blot for each cell line was plotted vs. relative glioma invasiveness. The lines through the data indicate the median for each invasiveness category; n = 8 highly/moderately invasive lines and n = 9 minimally/non-invasive lines; **indicates a statistical difference (one-tailed, unpaired t test) between the two categories of invasiveness at p<0.005. **C.** Human prostate tumor, breast tumor, astrocytoma, and GBM samples were examined by immunohistochemistry for total p120 catenin expression (using the 15D2 antibody), and Y228-phosphorylated p120 catenin expression. Bar: 100 µm.

Initially we used western blotting to evaluate the expression of Src, the overall level of SFK activation (using a Src Y416 phosphospecific antibody that cross-reacts with the analogous phosphotyrosine on other SFKs), and the downstream effector p120 catenin in a panel of GBM xenograft lines. Expression and phosphorylation levels were compared to the extent of orthotopic tumor invasion as evaluated by H&E staining of intracranial tumor sections. As seen in Figure 1A, there was a spectrum of Src activation across the xenograft lines evaluated. Interestingly, tumor lines (e.g. GBM6, GBM8, GBM26) with the highest level of phosphorylation of Y228 on p120-catenin were also the most invasive *in vivo*. In contrast, GBM10, which has modest levels of active Src but low levels of Y228 p120 phosphorylation, is not invasive when implanted orthotopically. Indeed, the level of p120 Y228 phosphorylation correlated directly with GBM invasiveness in the orthotopic xenografts (Figure 1B). We also evaluated the levels of Y228-phosphorylated p120 in tumor samples by immunohistochemistry (IHC). Generally, we observed that the levels of Y228-phosphorylated p120 were higher in tumor samples from patients with astrocytomas or GBMs than those from patients with breast or prostate cancer (Figure 1C). These data are consistent with previous studies suggesting that SFKs play an important role in mediating glioma tumor invasion [14]–[18], [20]. The data also highlight the significance of key SFK downstream effectors (i.e. p120 signaling).

**Figure 2:**
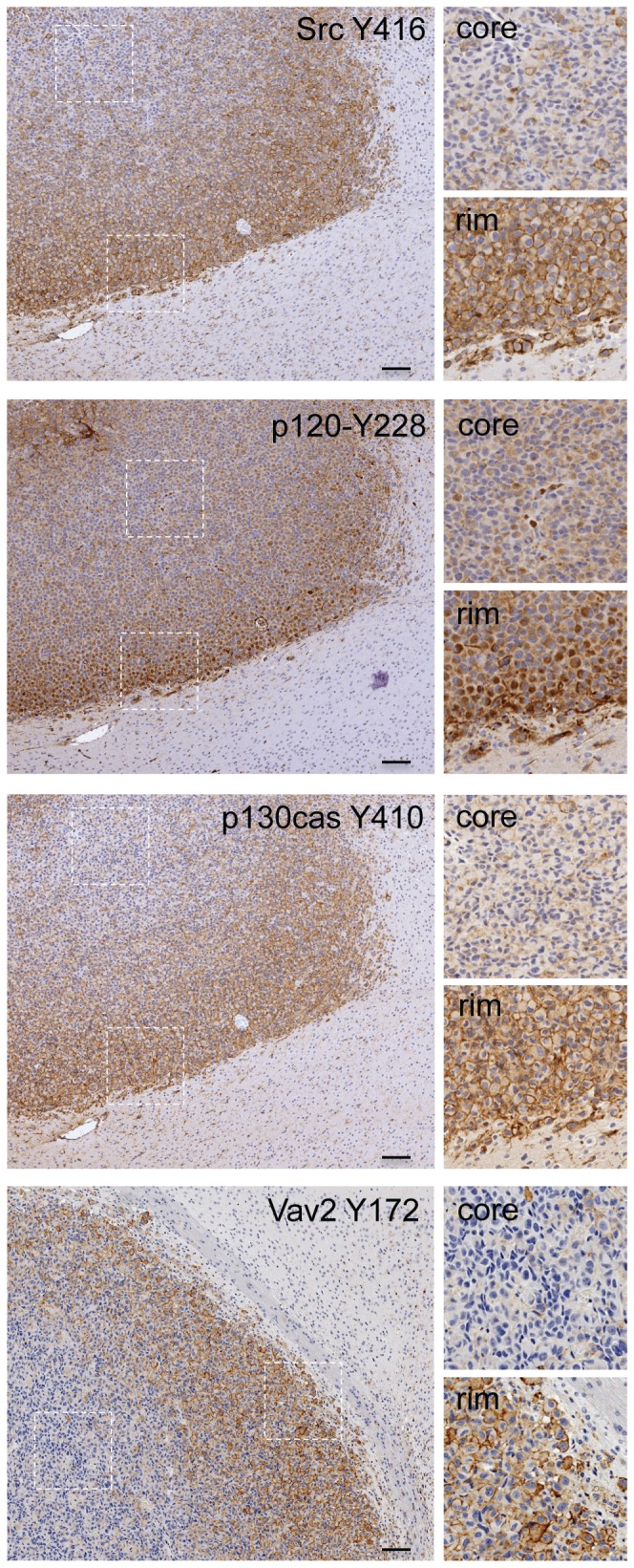
The invasive front of human GBM is a site of SFK activation. The spatial distribution of active SFK (as assessed by Y416-phosphorylated Src), Y228-phosphorylated p120 catenin, Y410-phosphorylated p130cas and Y172-phosphorylated Vav2 was assessed by immunohistochemistry of orthotopic GBM39 xenograft samples. Core indicates an image from the non-invading tumor core; rim indicates an image from the tumor's leading edge where the tumor interfaces with and can invade into the surrounding normal brain. Bar: 100 µm.

SFK-mediated signaling through the p120-catenin/Vav2 and the p130cas/DOCK180 pathways modulates Rho family GTPases, which supervise reorganization of the cytoskeleton to permit cell migration. To evaluate whether these signaling pathways are active in GBM, we used immunohistochemistry to evaluate the activation status of these SFK-dependent pathways in the non-invasive tumor core versus the invading tumor edge of orthotopically xenografted GBM39, which are responsive to SFK inhibition [19]. Consistent with a role of SFKs in driving tumor invasion, SFK activation (as assessed by Src Y416 phosphorylation) was dramatically higher at the leading/invading edge of the tumor (Fig. 2; rim denotes invasive edge) compared to the tumor core (Fig. 2; core). Likewise, phosphorylation of the direct SFK substrates p120-catenin, p130cas and Vav2 was observed at the invasive tumor edge but not in the tumor core. Together, the data indicate that the invasive front of human GBM is a site of SFK activation and suggest that SFKs target the p120/Vav2 and p130/DOCK180 signaling pathways to promote GBM cell migration and invasion.

**Figure 3:**
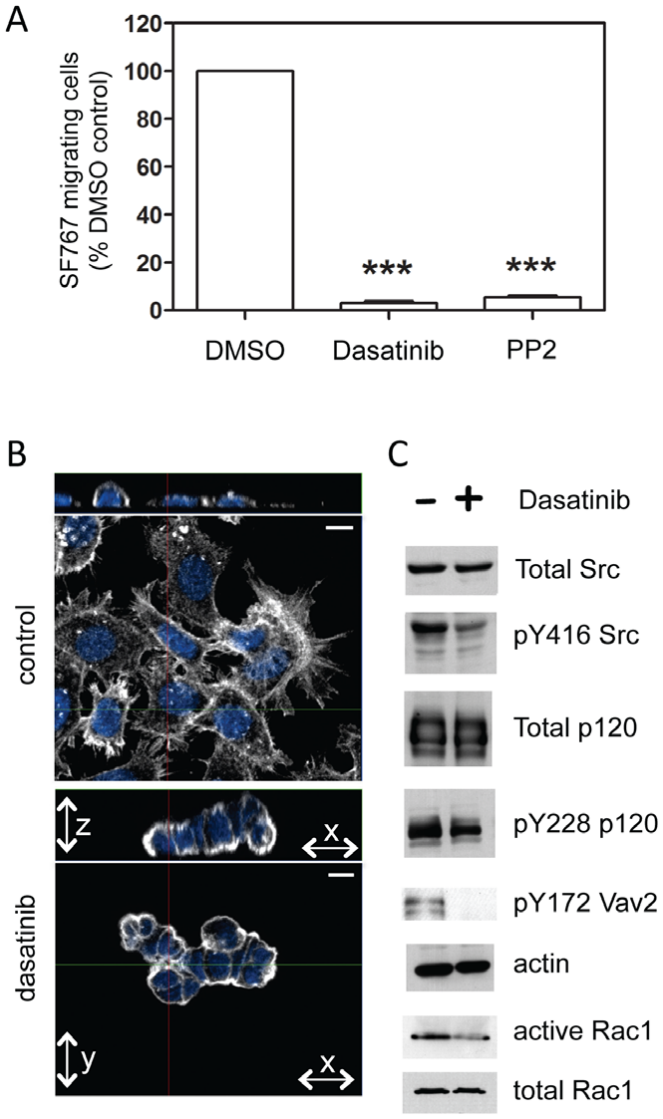
Dasatinib inhibits activation of SFKs and downstream targets in glioma. **A.** Migration of SF767 cells in the absence or presence of 10 µM dasatinib or 10 µM PP2 was determined using a trans-well migration assay. Inhibition of SFKs with dasatinib or PP2 resulted in fewer SF767 cells migrating toward the chemoattractant. The differences vs. control (DMSO) treatment are statistically significant (n = 4; one-way ANOVA with Dunnett's Multiple Comparisons post test; *** indicates p<0.0001). **B.** Immunofluorescent staining of actin (white) in SF767 cells after control (DMSO) treatment or treatment with dasatinib (10 µM for 24 hours). For each treatment, the top image is the x-z orientation and bottom is the x-y orientation. Blue staining is DAPI; scale bar is 10 µm. **C.** Whole cell lysates of SF767 cells treated for 24 hours with 10 µM dasatinib (+) or DMSO (−) were western blotted for total Src, Y416-phosphorylated Src, total p120-catenin, Y228-phosphorylated p120, Y172-phosphorylated Vav2, actin (as a loading control), or active or total Rac1.

The observation that SFK activation occurs at the leading tumor edge suggests that pharmacological inhibition of SFK signaling may be a particularly effective anti-invasive therapy for human GBM. To begin to address this, we treated conventional GBM cell lines with either the SFK inhibitor PP2 or dasatinib (BMS-354825; Sprycel®), a clinically-relevant ATP competitive multi-targeted kinase inhibitor that inhibits all SFK members, including Src, Lyn, Fyn and Yes [18], [21]. Both PP2 and dasatinib potently inhibited the directed migration of SF767 cells towards a gradient of FBS in transwell migration assays (Figure 3A). Similar results were obtained with the LN229 GBM cell line (data not shown). Interestingly, dasatinib treatment induced cytoskeletal rearrangement of SF767 cells leading to decreased cell spreading and compacted areas of cell-cell contact (Figure 3B). These effects of dasatinib were associated with decreased phosphorylation of Src at the Y416 activating site, p120 at Y228, Vav2 at Y172, as well as with reduced endogenous Rac1 activity (Figure 3C).

**Figure 4:**
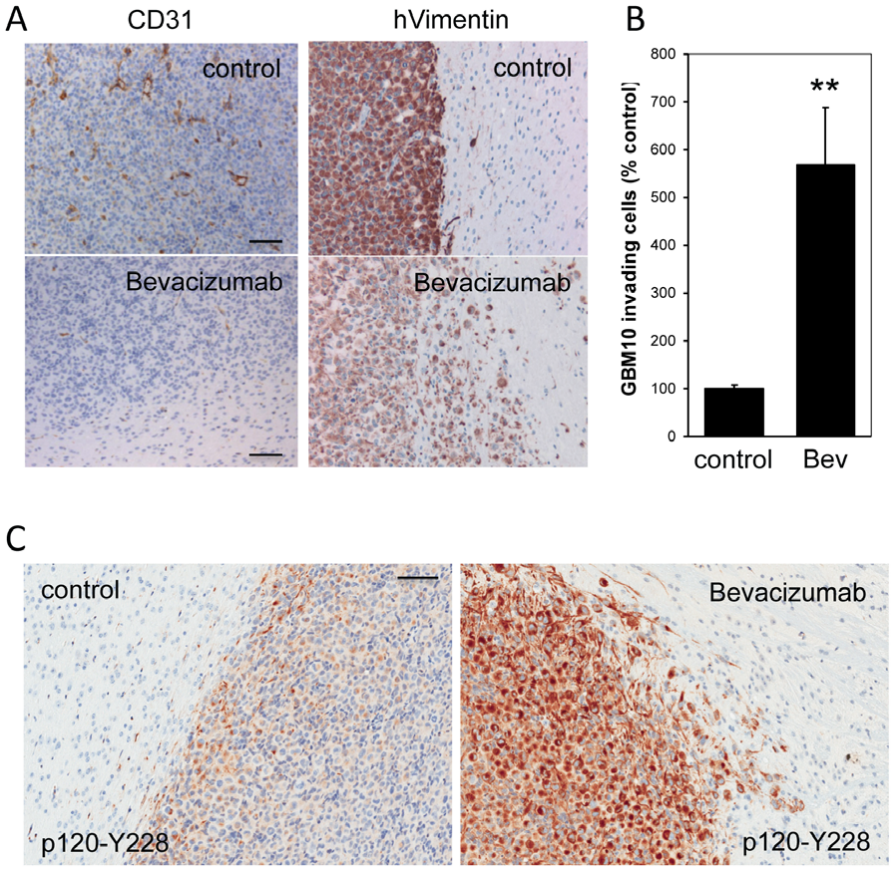
SFK signaling is associated with bevacizumab-induced GBM cell invasion. **A.** Orthotopically located GBM10 xenografts treated with or without bevacizumab were stained immunohistochemically for CD31 to show the location of tumor-associated blood vessels (left panels) or with antibody to human Vimentin to reveal the location of GBM10 tumor cells (middle panels). Bar: 100 µm. **B.** The number of single GBM10 cells invading into the surrounding normal brain parenchyma in control and bevacizumab-treated samples was counted in multiple fields and is plotted as percent of control treatment (right panel). The difference between the 2 treatments is statistically significant; ** represents p<0.001 (n = 18; Student's t-test). **C.** Immunohistochemistry was used to assess the extent of Y228-phosphorylated p120 catenin in orthotopically located GBM10 xenografts treated with or without bevacizumab. Bar: 100 µm.

Because tumor progression following anti-angiogenic bevacizumab therapy is often associated with increased cell invasion and co-option of normal vasculature, we next examined whether SFK signaling is involved in bevacizumab-induced GBM cell invasion. GBM10 tumor cells (a relatively non-invasive GBM xenograft line) were allowed to grow orthotopically in mice for two weeks and then the mice were treated with bevacizumab (5 mg/kg, twice a week, IP) for 19 days. Brains were then processed for immunohistochemistry. As expected, bevacizumab treatment abrogated neo-angiogenesis, as evaluated by CD31 staining of tumor associated blood vessels (Figure 4A). Importantly, upon bevacizumab treatment single tumor cells invading the brain parenchyma could be readily identified (GBM10 cells were identified using an antibody that recognizes human but not mouse vimentin). Indeed, there was a statistically significant increase in the number of invading single tumor cells with bevacizumab treatment compared to control treatment (Figure 4B). Relevant to our initial hypothesis, the increased invasion of the bevacizumab-treated GBM10 orthotopic tumors was associated with a significant increase in p120 phosphorylation at the Y228 SFK-target site (Figure 4C). The data indicate that GBM10 is an appropriate animal model of bevacizumab-induced invasiveness, and show that increased Y228 phosphorylation of p120 correlates with the invasiveness of bevacizumab treated tumors.

**Figure 5:**
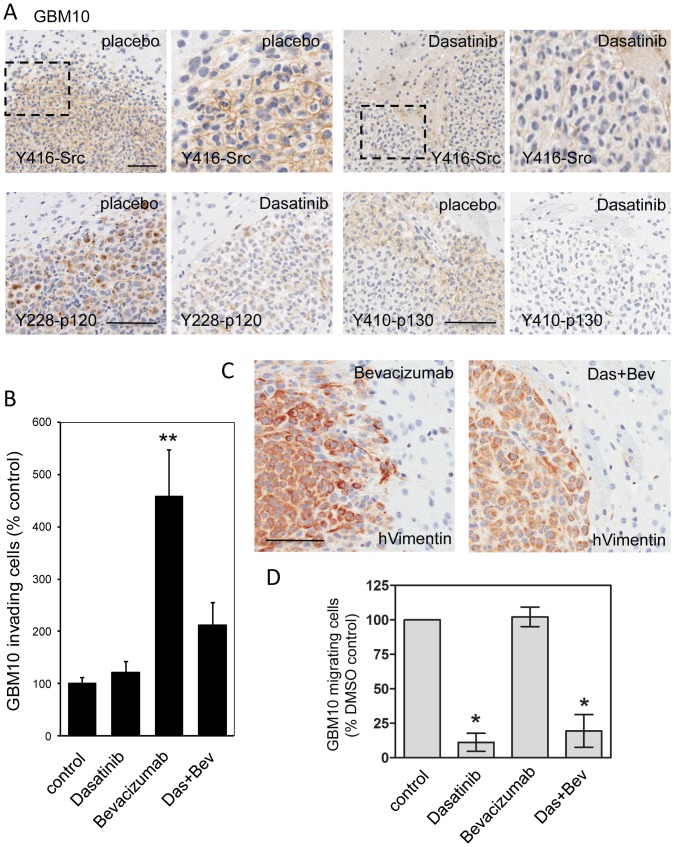
Dasatinib suppresses SFK signaling and bevacizumab-induced GBM cell invasion. **A.** Orthotopically located GBM10 xenografts treated with or without dasatinib were stained immunohistochemically for Y416-phosphorylated Src, Y228-phosphorylated p120, and Y410-phosphorylated p130cas. Upper panels: Left panels are lower magnification; right panels are higher magnification of boxed inset. Bar: 100 µm. **B.** The number of single GBM10 cells invading into the surrounding normal brain parenchyma in placebo- (control), dasatinib-, bevacizumab-, and dasatinib+bevacizumab-treated samples was counted in 18 unique fields from at least 3 separate mice per treatment. Data are expressed as % control and represent the mean ± SD (*n* = 18; ** represents p<0.001, ANOVA with Dunnett's Multiple Comparisons post test). **C.** Orthotopically located GBM10 xenografts treated with bevacizumab with or without dasatinib were stained immunohistochemically for human Vimentin to show the localization and invasion pattern of the GBM10 tumor cells. Bar: 100 µm. **D.** Migration of GBM10 cells in the absence or presence of 10 µM dasatinib, 0.5 mg/ml bevacizumab, or dasatinib+bevacizumab was determined using a trans-well migration assay. Inhibition of SFKs with dasatinib alone or in combination with bevacizumab resulted in fewer GBM10 cells migrating toward the chemoattractant; bevacizumab treatment alone had no effect on GBM10 migration. * indicates a statistically significant difference vs. control treatment (n = 3; one-way ANOVA with Dunnett's Multiple Comparisons post test; * indicates p<0.05).

Based on the above data, we hypothesized that suppressing SFK activities with dasatinib would block the invasive phenotype of bevacizumab-treated GBM10 cells. Dasatinib has partial blood-brain barrier penetration, but it exhibits relatively poor pharmacokinetics (short half life of <4 h; [42]). To determine whether dasatinib can suppress SFK activity *in vivo*, GBM10 tumor cells were grown orthotopically in mice for two weeks and then the mice were treated for 19 days with 30 mg/kg dasatinib (twice daily, PO). Mice were sacrificed 2 hours after the last dasatinib injection and the extent of SFK activation in the orthotopic xenografts was determined immunohistochemically. Figure 5A shows that dasatinib treatment inhibited the phosphorylation of SFKs at the conserved Y416 SFK activation site under these conditions. Moreover, similar to the SF767 data (Figure 3), dasatinib suppressed the phosphorylation of p120 at Y228 and p130cas at Y410 in GBM10 xenografts (Figure 5A). Collectively, the data argue that increased SFK activity is associated with an invasive phenotype in GBM xenografts and that dasatinib can block both SFK activation and downstream signaling events in an animal model of human GBM.

Finally, we examined the effect of dasatinib on the invasiveness of bevacizumab-treated GBM10 orthotopic xenografts *in vivo*. GBM10 tumor cells were grown orthotopically for two weeks and then the mice were treated for 19 days with placebo, bevacizumab (5 mg/kg, twice a week, IP) and/or dasatinib (30 mg/kg, twice daily, PO). Brains were then excised and, along with brain sections from the vehicle control, bevacizumab-only and dasatinib-only groups, processed for IHC to human vimentin. The relative invasiveness of GBM10 tumor cells was not affected by dasatinib treatment alone, but the increased invasiveness of bevacizumab-treated GBM10 cells was largely blocked by co-treatment with dasatinib (Figure 5B). Indeed, dasatinib suppressed the diffuse nature of bevacizumab-treated GBM10 tumor margins (Figure 5C), reverting them back to the well-defined, non-invasive border seen in control GBM10 xenografts (compare Figure 4A and 5C). To parse the role of the tumor microenvironment in the response of the GBM10 cells to bevacizumab and dasatinib, we utilized short-term culture of isolated GBM10 cells. GBM10 cells were seeded in transwells and allowed to migrate towards a gradient of FBS for 20 hours in the presence or absence of dasatinib (10 µM) and/or bevacizumab (0.5 mg/ml). While bevacizumab had no effect on the migration of GBM10 cells, dasatinib markedly reduced cell migration (Figure 5D). The data indicate that dasatinib acts directly on the tumor cells to suppress invasion, and strongly suggest that bevacizumab affects cell migration by regulating the tumor microenvironment.

## Discussion

The lethality of human gliomas is associated with tumor cell invasion, which limits the efficacy of surgical and radiological therapies. Previous studies have shown that signaling downstream of SFKs, including activation of p120 catenin, p130cas, and their resultant Rac1 signaling, can promote tumor cell migration and invasion. Using an orthotopic xenograft model of GBM, we show here that the invasive behavior of human gliomas is closely related to the phosphorylation of p120 catenin at the SFK phosphorylation site Y228. Moreover, SFK activation and phosphorylation of key downstream targets, including p120 and p130cas, are upregulated at the leading edges of orthotopic glioma xenografts.

Dasatinib is a potent oral, ATP-competitive, multi-targeted kinase inhibitor that inhibits Bcr-Abl and all members of the Src family of kinases (SFK), including C-Src, Lyn, Fyn and Yes [18], [21]. At higher concentrations, dasatinib also inhibits c-kit, and PDGFR [21]. Studies in cultured cells as well as animal models of glioma [16], sarcoma [43], colon [44], lung [45], head and neck [45], pancreatic [46] and prostate [47], [48] cancers have shown that dasatinib can potently suppress tumor cell migration and invasion. A related SFK inhibitor (bosutinib) was also shown to suppress the migration and invasion of breast [49], [50] and colon [51], [52] cancer cells *in vitro* and *in vivo*, further validating the use of SFK inhibitors to block tumor cell invasion. In agreement with the published literature, we find that suppression of SFK activity with dasatinib in SF767 glioma cells inhibits p120 Y228 phosphorylation, Vav2 Y172 phosphorylation, and Rac1 activation, and blocks their directed migration in culture. Taken together, our data suggest that SFK inhibition in general, and dasatinib treatment in particular, should be further evaluated for the treatment of primary human gliomas.

Preclinical studies universally suggest that increased tumor invasiveness is a major impediment to the efficacy of anti-angiogenic GBM therapy (for review see [53]). Several clinical studies are consistent with this observation [11], [53]–[55], but not all [56], highlighting a) the difficulty of establishing tumor cell infiltration radiologically under conditions of vascular normalization (bevacizumab), and b) the need for follow-up observation of brain tissue upon autopsy [54]. Our studies with GBM10 xenografts are consistent with prior preclinical data and highlight the importance of SFK activation in the bevacizumab-mediated induction of GBM10 cell invasion. The data support the administration of dasatinib in combination with bevacizumab containing regimens, since dasatinib could counteract the bevacizumab-associated increase in glioma cell invasion, a likely key mechanism of bevacizumab resistance.

The mechanism by which SFK activity is increased at the leading edges of human gliomas is currently unclear. One possibility is activation due to local hypoxia. A number of studies suggest that in addition to a central anoxic and often necrotic tumor core, hypoxic conditions exist at the tumor periphery, where hypoxia-induced, localized expression of VEGF contributes to neo-angiogenesis [48], [49], [50], [51]. This mechanism is targeted by anti-angiogenic therapy such as bevacizumab, which is rapidly becoming the treatment of choice for recurrent GBM but is often associated with a diffuse or multi-focal pattern of recurring tumors. Src can be activated by tumor hypoxia [18], [24] in a mitochondrial reactive oxygen species and NADPH oxidase dependent manner [57], [58], and is associated with increased tumor cell migration and invasion. We postulate that anti-angiogenic agents like bevacizumab may cause a selective loss of neo-vasculature at the invading front, prolonging locally hypoxic conditions that then induce SFK activation, tumor cell migration and invasion [59], [60]. An alternative, hypoxia-independent mechanism of SFK activation may involve the compensatory activation of c-Met signaling upon VEGF suppression with bevacizumab [61]. Regardless the mechanism, both models are consistent with the increased p120 Y228 phosphorylation at the invasive front of bevacizumab-treated orthotopic xenografts, and the ability of dasatinib to effectively block the invasiveness of bevacizumab-treated GBM10 orthotopic tumors.

The data argue strongly that SFK activation underlies the increased migration/invasion of human gliomas treated with bevacizumab. The data further suggest that dasatinib and/or other SFK inhibitors could block bevacizumab-induced glioma invasion and support testing the combination of dasatinib with bevacizumab in the clinic to delay the development of resistance to bevacizumab. Following phase I dose escalation, which demonstrated excellent tolerance of this regimen, a phase II trial of bevacizumab + dasatinib versus placebo in recurrent glioma patients is currently accruing patients through the Alliance for Clinical Trials in Oncology cooperative group.

## Materials and Methods

### Ethics Statement

*Animal studies were approved by the Mayo Clinic Institutional Animal Care and Use Committee (IACUC) and were conducted according to Mayo Clinic IACUC guidelines for animal husbandry.*

### Orthotopic xenograft model: evaluation of relative invasiveness

Each of the 17 serially passaged xenograft cell lines used in this study are derived from resected tumors from different human patients and are propagated by serial transplantation in the flank of nude mice [41]. Tumors are propagated exclusively *in vivo* in order to preserve molecular and histopathologic features of the primary patient tumor specimens. All xenograft cell lines have been described previously [41], [62]–[65]. The relative invasiveness of the lines was assessed in a previous publication [65]. Briefly, the degree of invasiveness was evaluated by H&E examination of sections of mouse brain containing orthotopically injected xenograft lines. In tumors that were classified as “highly invasive”, tumor cells could be easily identified extending along the commissural structures (corpus callosum and anterior commissure) to the opposite hemisphere. In tumors that were considered “minimally invasive” or “non invasive” a “nearly sharp” border could be identified between the tumor mass and surrounding brain parenchyma, with minimal or no intermixing of tumor cells with surrounding parenchyma at the edge of the tumor. “Moderately invasive” xenografts showed intermediate features, with cells extending well away from the main tumor mass, but remaining largely in the ipsilateral hemisphere.

### Orthotopic xenograft model: evaluation of drug treatment on tumor invasiveness

Short-term explant cultures of GBM10 cells derived from flank tumor xenografts were injected orthotopically into nude mouse brain as described [66]. Cells were grown orthotopically for two weeks, and then the mice were treated with placebo, bevacizumab (5 mg/kg, twice a week, IP), dasatinib (30 mg/kg, twice daily, PO) or bevacizumab+dasatinib. Mice were euthanized after 19 days of treatment; if part of a dasatinib-treatment group, euthanasia was done 2 hours after the last dasatinib injection. Brains from these mice were formalin-fixed, and paraffin-embedded. Tissue samples were then processed for routine H&E or immunohistochemistry. Tumor cell invasion was determined by a blinded observer by quantifying the number of invading cells on sections of mouse brains stained with an anti-human vimentin antibody to selectively identify human tumor cells. A combination of user interaction and automatic thresholding (using ImageJ software) was used to delineate: 1) the solid tumor core, 2) the rim (edge) of solid tumor tissue, and 3) the location of individual tumor cells infiltrating normal brain. The number of individual cells crossing the solid tumor rim was counted in multiple fields of equivalent size and tumor position and results were grouped and expressed as percent control (invasion in the placebo group).

### Immunohistochemistry

H&E and immunohistochemical staining presented in Figures 1, 2, 4, and 5 was performed by the Mayo Clinic Florida Cancer Biology Histology Resource using standard methods. Antibodies were as follows: anti-p120 catenin (clone 15D2, Zymed/Invitrogen, Grand Island, NY), anti-phospho Y228 p120 catenin (BD Biosciences, San Jose, CA), anti-phosphoY416 Src (#2101, Cell Signaling Technology, Danvers, MA), anti-phosphoY410 p130Cas (Cell Signaling Technology), anti-CD31 (Santa Cruz Biotechnology, Santa Cruz, CA), and anti-vimentin (#M7020, Dako North America, Carpinteria, CA).

### Conventional and short-term xenograft cell culture

GBM10 cells were harvested from flank xenografts for short term culture as described [66]. SF767 were obtained from the UCSF/Neurosurgery Tissue Bank, San Francisco, CA. SF767 cells and GBM10 cells were cultured on tissue-culture treated plastic dishes at 37°C, 5% CO_2_, in DMEM media containing 10% Fetal Bovine Serum (not heat-inactivated), an additional 2 mM L-glutamine, and 1% non-essential amino acids. Additionally, penicillin and streptomycin was added to the GBM10 culture media.

### Preparation of whole cell lysates for western blot

#### Flank xenograft tissue lysates

Flash frozen flank xenograft tissues were homogenized at 0°C in SDS Lysis buffer (2% w/v SDS, 4 M deionized urea, 62.5 mM Tris-Cl pH 6.8, 1 mM EDTA, 5% v/v ß-Mercaptoethanol, with 1 mM Na_3_VO_4_, 50 mM NaF, and 1 mM PMSF). Lysates were then cleared by centrifugation at 13000 rpm at 4°C for 20 minutes. Total protein in the supernatants was quantified using the nitric acid method [67], while the remaining lysates were mixed with Laemmli Sample Buffer (2× final concentration, 0.1 M Tris-Cl pH 6.8, 2% SDS, 10% sucrose, 0.24 M ß-Mercaptoethanol, 0.008% bromophenol blue) and boiled for 5 minutes before being analyzed by Western Blot.

#### SF767 cell lysates

Whole cell lysates were made by lysing the cells in ice cold RIPA buffer (50 mM Tris-Cl pH 7.4, 150 mM NaCl, 1% Igepal CA-630 (NP-40 substitute), 0.5% Deoxycholic Acid, 0.1% SDS, with 1 mM Na_3_VO_4_, 1 mM EDTA, 50 mM NaF, 1 mM PMSF, 5 µg/ml leupeptin, and 2 µg/ml aprotinin) for 7 minutes, followed by brief sonication. Ten µl samples of each lysate were quantitated for total protein using the BioRad Protein Assay Dye Reagent (BioRad Laboratories, Hercules, CA). Remaining lysates were mixed with Laemmli Sample Buffer (2× final concentration) and boiled for 5 minutes before being analyzed by Western Blot.

### Active Rac1 assay

Rac1 activity was determined in SF767 cells using a specific pull-down assay for the activated form of Rac1 as reported previously [68]. PAK-1 PBD (Upstate Biotechnology) bound to glutathione agarose beads was used to precipitate GTP-bound Rac1 from cell lysates. Active, GTP-bound Rac1, as well as total Rac1, were visualized by SDS-PAGE and Western blotting using a Rac1-specific mAb (BD Biosciences).

### Western blotting

Equal µg amounts of protein lysates were separated by SDS-PAGE and transferred to nitrocellulose filters using standard methods. For detection of standard (non-phospho) proteins, blots were blocked in 5% nonfat dry milk in Tris-buffered saline (TBS), pH 7.4 before being incubated in primary antibodies in 5% milk/TBS. Blots for phospho-proteins were blocked in 5% milk plus 5% BSA in TBS+0.1% Tween 20 (TBST) before being incubated with primary antibodies in 5% BSA/TBST. Blots were rinsed three times and washed four times 5 minutes in TBST. Blots were then incubated with HRP-conjugated secondary antibodies in 5% milk/TBS (non-phosho proteins) or 5%milk/5% BSA/TBST (phospho-proteins), and rinsed and washed in TBST as before. Proteins were detected using Amersham ECL Western Blotting Detection Reagents (GE Healthcare, Buckinghamshire, United Kingdom). Primary antibodies were as follows: anti-Src (clone 32G6, Cell Signaling Technology), anti-phosphoY416 Src (#2101, Cell Signaling Technology), anti-phospho Y228 p120 catenin (Epitomics, Burlingame, CA), anti-p120 catenin (clone 15D2, Zymed/Invitrogen), anti-actin (A2066, Sigma-Aldrich, St. Louis, MO), anti-phosphoY172 Vav2 (Santa Cruz Biotechnology), anti-Rac1 (BD Biosciences). HRP-conjugated secondary antibodies (anti-mouse and anti-rabbit) were obtained from Jackson ImmunoResearch Laboratories, West Grove, PA.

### Immunofluorescence

SF767 cells were plated on glass coverslips and allowed to adhere for 24 hours. Cells were then treated for 24 hours with 10 µM dasatinib or a 1∶1000 dilution of DMSO (vehicle control) in media. Cells were fixed with 3% paraformaldehyde at room temperature for 30 minutes, washed twice in PBS+10 mM glycine, permeabilized with PBS/0.2% Triton X-100 for 2.5 minutes at room temperature, then washed again with PBS/glycine before blocking. Cell nuclei and F-actin were labeled by incubation with DAPI and Alexa 594-conjugated phalloidin (Invitrogen), respectively. Coverslips were mounted on glass slides with Aqua Poly/Mount (Polysciences, Inc. Warrington, PA). Cells were visualized on a Zeiss LSM 510 META laser scanning confocal microscope (Carl Zeiss Microscopy, Thornwood, NY) using a 63× Plan-Apochromat 63×/1.4 oil objective. Z-stack images (1 µm section thickness) were acquired with Zeiss AIM software at 51.2 µsec pixel time scan speed and compiled in Adobe Photoshop.

### Transwell migration assay

Transwell migration of SF767 or GBM10 cells was assessed as described previously [65]. Briefly, 1×10^5^ serum-starved cells were plated in collagen-coated transwell culture inserts (upper chamber) (Nalge Nunc International, Rochester, NY) and placed in 24 well plates containing DMEM/BSA with 0.5% Fetal Bovine Serum (lower chamber). Cells were allowed to migrate to the underside of the transwell for 6 hours (SF767 cells) or 19 hours (GBM10 cells) at 37°C, 5% CO_2_. For assays that included a drug treatment, the drug was included in both the upper and lower chambers. Cells on the underside of the transwell chambers were collected by incubating in Cell Dissociation Buffer (0.25% phenol red-free Trypsin EDTA (Invitrogen) in Hank's Balanced Salt Solution) for 20 minutes at 37°C, 5% CO_2_. The number of cells was quantified using the CyQuant Cell Proliferation Assay kit (Invitrogen) as described previously [65]. This method measures DNA concentration to determine the relative number of cells in each migration condition. Data are expressed as the percentage of control (DMSO vehicle-treated) cell migration and are presented as the mean +/− SEM of 4 independent experiments performed in triplicate.
